# A stereotactic frame-based drill guide-aided setting for laser interstitial thermal therapy

**DOI:** 10.1007/s00701-021-04869-1

**Published:** 2021-05-13

**Authors:** Markus F. Oertel, Lennart H. Stieglitz, Oliver Bozinov

**Affiliations:** 1 Department of Neurosurgery, University Hospital Zurich, Frauenklinikstrasse 10, 8091 Zurich, Switzerland; 2 Clinical Neuroscience Center, University Hospital Zurich, Frauenklinikstrasse 10, 8091 Zurich, Switzerland; 3 Department of Neurosurgery, Cantonal Hospital St. Gallen, Rorschacher Strasse 95, 9007 St. Gallen, Switzerland

**Keywords:** Ablation, Interstitial thermal therapy, Laser, Stereotactic frame, Visualase

## Abstract

**Background:**

Magnetic resonance imaging (MRI)-guided laser interstitial thermal therapy (MRIgLITT) was demonstrated to be a viable neurosurgical tool. Apart from its variety of indications, different operative and technical nuances exist. In the present report, for the first time, the use and ability of a traditional Riechert-Mundinger (RM) stereotactic system combined with a novel drill guide kit for MRIgLITT are described.

**Methods:**

A stereotactic frame-based setting was developed by combining an RM system with a drill guide kit and centering bone anchor screwing aid for application together with an MRIgLITT neuro-accessory kit and cooled laser applicator system. The apparatus was used for stereotactic biopsy and consecutive MRIgLITT with an intraoperative high-field MRI scanner in a brain tumor case.

**Results:**

The feasibility of an RM stereotactic apparatus and a drill guide kit for MRIgLITT was successfully assessed. Both stereotactic biopsy and subsequent MRIgLITT in a neurooncological patient could easily and safely be performed. No technical problems or complications were observed.

**Conclusion:**

The combination of a traditional RM stereotactic system, a new drill guide tool, and intraoperative high-field MRI provides neurosurgeons with the opportunity to reliably confirm the diagnosis by frame-based biopsy and allows for stable and accurate real-time MRIgLITT.

## Introduction

Magnetic resonance imaging (MRI)-guided laser interstitial thermal therapy (MRIgLITT) is an inviting treatment option in neurosurgery. Using a stereotactically inserted laser applicator, patients can be treated through an elegant and minimally invasive approach.

At present, many clinical indications, surgical variations, and technical nuances for MRIgLITT exist [1, 2, 4, 5, 7]. Here, for the first time, a traditional Riechert-Mundinger (RM) stereotactic system [6] was implemented in combination with a novel drill guide kit for MRIgLITT. Based on a neurooncological case, the setting and procedure are described in detail and a discussion of its potential is provided.

## Case presentation

**Fig. 1:**
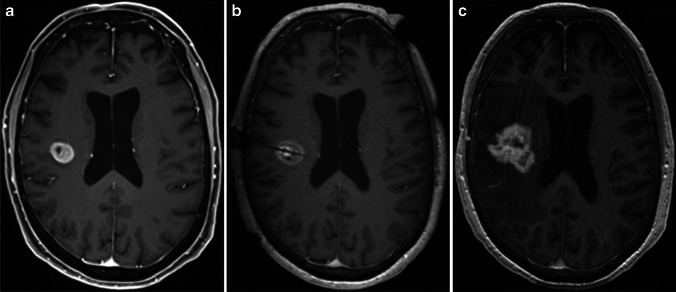
Axial magnetic resonance imaging T1-weighted sequences demonstrating a diffuse contrast-enhancing lesion accompanied by perifocal edema within the right parietal operculum consistent with a high-grade glioma. **a** Preoperative at presentation. **b** Intraoperative after ablation with the laser probe inserted along the tumor extent. **c** Postoperative 16 months following surgery and adjuvant combined radio-chemotherapy revealing tumor progression

A 65-year-old female presented to our hospital complaining of intermittent left-sided hemidysesthesia 11 days prior to admission. Additionally, she had a history of obstructive sleep apnea syndrome, chronic venous insufficiency, hypertensive cardiac disease, coronary sclerosis, hyperuricemia, dyslipidemia, hypothyroidism, osteoarthritis, and glaucoma. Upon admission, she underwent comprehensive clinical assessment whereby the neurological examination revealed mild hypesthesia of the left half of the body. Cranial MRI demonstrated a diffuse contrast-enhancing lesion accompanied by perifocal edema within the right parietal operculum consistent with a high-grade glioma (Fig. 1a).

MRIgLITT was discussed between disciplines and offered to the patient as an alternative to open microsurgery. The indication for the procedure was based on multiple factors including the increased perioperative risk with reduced patient health status, multiple concomitant diseases, and the anatomical deep-seated subcortical tumor location. Written informed consents for surgery and general research for collection of clinical data and use for academic or educational purposes were obtained from the patient. Local Review Board approval was not needed for this single report, as patient data was de-identified in compliance with institutional requirements.

The patient underwent stereotactic biopsy with frozen sections and subsequent MRIgLITT for ablation of the right parietal lesion (Fig. 1b) as described in detail below. The histopathological examination confirmed the diagnosis of a glioblastoma grade IV according to the World Health Organization (WHO) Brain Tumor Classification System [9]. The patient was discharged on the fourth postoperative day. She received adjuvant combined radio-chemotherapy with concomitant alkylating agent temozolomide treatment according to the decision of the institutional interdisciplinary neurooncological board. The further clinical course was uneventful with remittent sensory deficits. Only after 16 months following surgery, routine MRI examinations showed signs highly suspect of tumor progression (Fig. 1c). This was clinically accompanied by mild left-sided hemiparesis whereupon due to the tumor growth under temozolomide therapy angiogenesis inhibition by bevacizumab was initiated.

## Stereotactic frame-based drill guide-aided magnetic resonance imaging-guided laser interstitial thermal therapy procedure

In the present report, an MRI-guided cooling and laser applicator system and neuro-accessory kit (Visualase®; Medtronic Navigation Inc., Louisville, CO, USA) for soft tissue ablation in open, percutaneous, and interstitial surgical procedures was used. The complete MRIgLITT setting comprises a targeting and insertion system, the laser ablation kit, and an intraoperative MRI scanner. All of the technology employed was United States of America (USA) Food and Drug Administration (FDA) and European Medicines Evaluation Agency European Union (EU) Conformité Européenne (CE) marketing approved for the purposes described. Prior to the first application in a patient, an unproblematic separate dry run with MRIgLITT in a human artificial gel-filled head model (Medtronic Inc., Minneapolis, MN, USA) was performed.

The patient underwent 3-Tesla (T) MRI (Magnetom® Skyra VD13; Siemens Healthcare GmbH, Erlangen, Germany) examination for diagnostic workup and stereotactic planning prior to surgery. Images were transferred to the StealthStation® S7® Surgical Navigation System (Medtronic Inc., Minneapolis, MN, USA). Optimal entry site, transcortical and perlesional catheter placement trajectory, and target were then determined, taking safe accessibility and ideal ablation coverage into account.

The day of surgery, the patient received general anesthesia and oral intubation. After standardized team time out and safe surgery check with the patient in supine position, administration of intravenous single-dose antibiotics (1.5 g cefuroxime), and skin disinfection, the RM headframe was mounted with four screws under sterile conditions and provided with a CT localization set consisting of four fiducial plates (Rev. A; Inomed Medizintechnik GmbH, Emmendingen, Germany). A contrast-enhanced CT (Somatom® X.cite; Siemens Healthcare GmbH, Erlangen, Germany) examination for frame-based anatomical referenciation was obtained. CT images were automatically stereo-localized by the Medtronic neuronavigation system and computationally fused with the preoperative MRI images for final planning. Following thorough adjustment of the coordinates, the RM stereotactic bow was mounted on the ring.

**Fig. 2:**
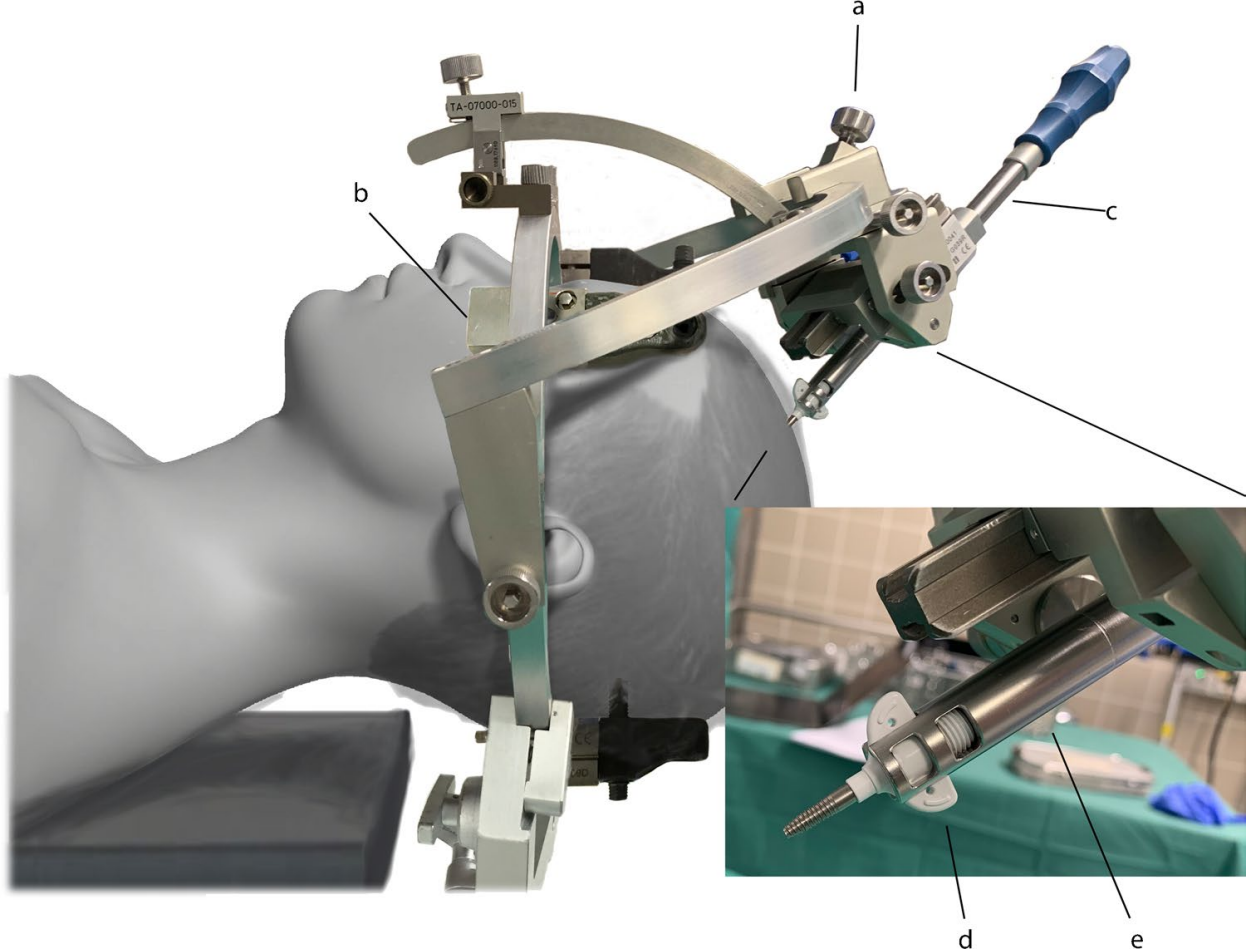
Schematic three-dimensional diagram with incorporated photograph of the specialized targeting and insertion system with a traditional Riechert-Mundinger apparatus and the novel drill guide kit. The stereotactic system including semicircular aiming bow with fixed instrument holder (a) and circular base ring (b) is combined with the drill guide kit (c) and mounted on a human manikin for placement of a titanium bone anchor (d) via the centering screwing aid (e) and subsequent fixation of the laser applicator

**Fig. 3:**
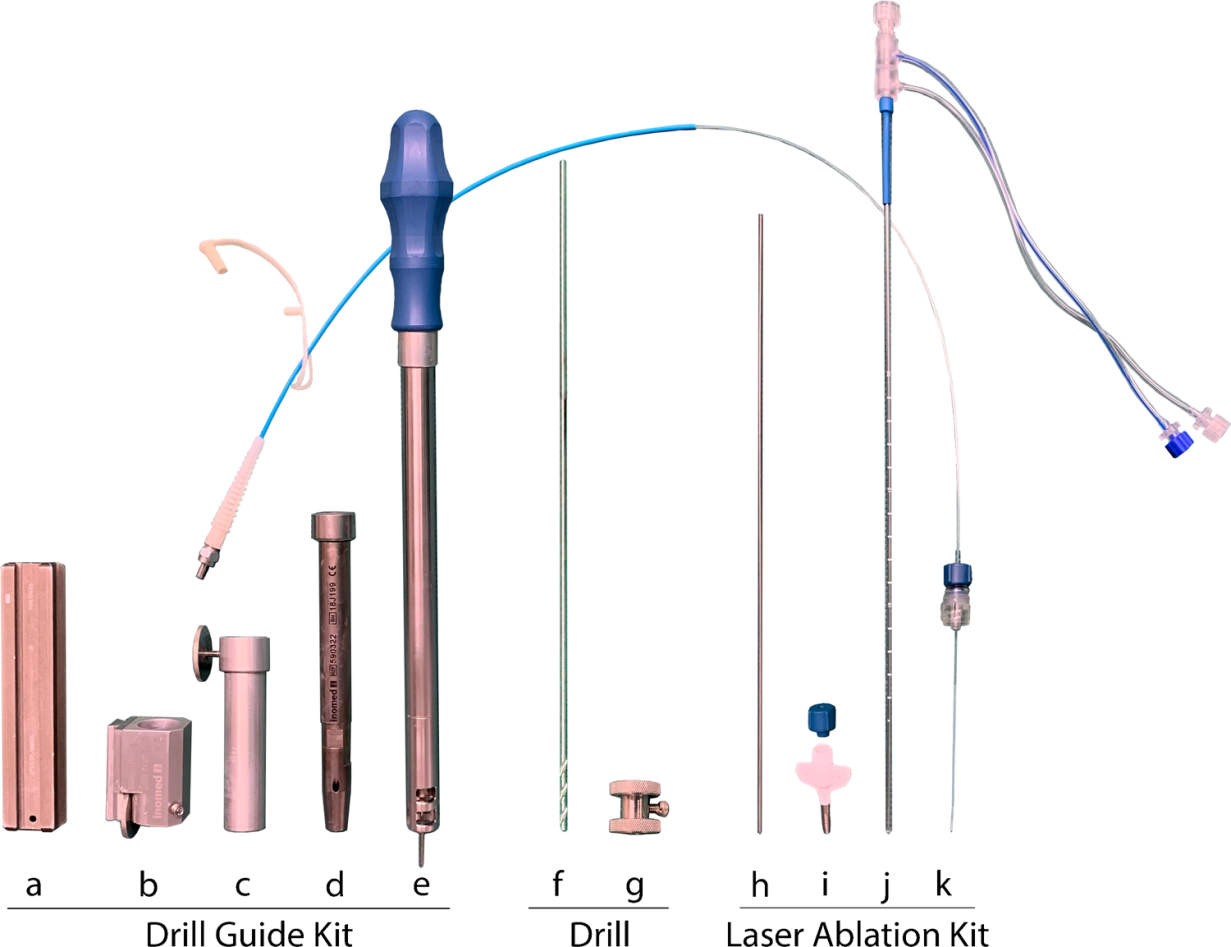
Schematic three-dimensional diagrams of the dedicated insertion and ablation equipment applied in the novel stereotactic frame-based and drill guide-aided setting for brain biopsy, laser applicator placement, and magnetic resonance imaging-guided interstitial thermal therapy. Insertion kit including a dove tail rail (a), sleeve (b), guide (c), reducing tube (d), screwing aid (e), twist drill (f), and clumping screw (g). Laser ablation kit comprising an alignment rod (h), bone anchor (i), applicator (j), and laser fiber (k)

The targeting and insertion system (Figs. 2 and 3) consists of an RM stereotactic circular CT-adapted titanium or half-open MRI compatible ceramic base ring, a semicircular aiming bow, a fixed instrument holder, a localization set available for CT and MRI applications, and a target point simulator (Inomed Medizintechnik GmbH, Emmendingen, Germany) combined with a drill guide kit and bone anchor centering screwing aid (Inomed Medizintechnik GmbH, Emmendingen, Germany) along with a StealthStation® S7® planning station with software (Medtronic Inc., Minneapolis, MN, USA). The drill guide kit enables stereotactic-based drilling with a Colibri II handpiece (DePuy Synthes, Raynham, MA, USA) and a 3.2-mm diameter and 30-cm length twist drill with a clumping screw (AD-TECH Medical Instrument Corporation, Oak Creek, WI, USA) for insertion of a brain biopsy cannula of 2.1-mm diameter (Neuromedex GmbH, Hamburg, Germany) and a bone anchor (Medtronic Inc., Minneapolis, MN, USA) thereafter. The kit consists of a 3-piece (sleeve, guide with knurled screw, reducing tube) drill guidance of 3.2-mm inner diameter, 19.5-mm outer diameter, and a length of 139 mm, a bone anchor centering screwing aid of 14-mm outer diameter and a length of 314.7 mm with hand grip, and a dove tail rail of 120-mm length for fixation on the RM bow.

After skin disinfection, sterile draping, and confirmation of the entry point, a 4-mm stab incision with a surgical scalpel blade no. 11 was performed and the reducing tube was placed into the guidance to accommodate the drill. Once the craniostomy was created, the dura was perforated with a stiffening stylet with a length of 35 cm and a diameter of 1.6 mm (Visualase®; Medtronic Navigation Inc., Louisville, CO, USA), and a histopathological sampling with the side-cut brain biopsy cannula was performed.

The laser ablation kit used (Fig. 3) includes a titanium bone anchor, light source, laser fiber, 10-mm applicator tip, pump tubing set, and tubing extension. Before insertion, the integrity and functionality of the laser catheter were controlled, and the planned depth measured. To insert the laser applicator in the center of the target area, the drill guidance without the reducing tube was used to pass the centering screwing aid for fixation of the bone anchor with a length of 4.1 cm and an outer diameter of 3.2 mm into the calvarian burr hole along the proper trajectory. This holds the laser applicator provided with a blue cap of 0.3-cm length. The sufficient passage of the anchor was controlled by an alignment rod of 26.7-cm length and diameter of 1.6 mm (Visualase®; Medtronic Navigation Inc., Louisville, CO, USA). Once in place, the RM bow was removed, and the catheter was passed to the appropriate depth. Upon reaching the target, it was secured to the bone anchor by tightening the cap. Subsequently, the RM frame and screws were removed.

**Fig. 4:**
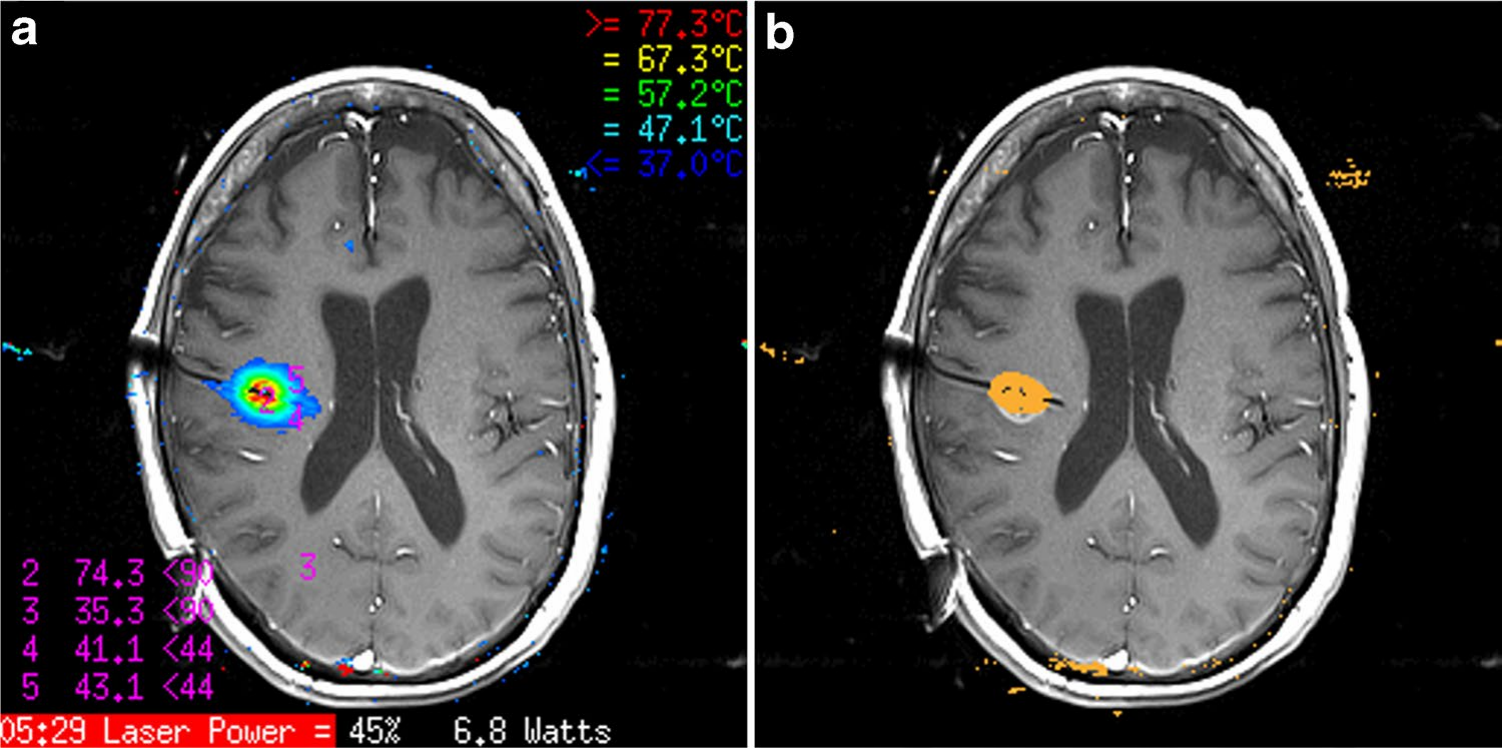
Images of tumor ablation after stereotactic frame-based and drill guide-aided brain biopsy and laser probe insertion. **a** Axial contrast-enhanced real-time magnetic resonance imaging for thermometry. **b** Monitoring of thermal damage during the magnetic resonance imaging-guided interstitial thermal therapy procedure with a 10-mm fiber core laser applicator

Intraoperative high-field MRI was performed using an 8-channel head coil (NORAS MRI products, Hoechberg, Germany) in a 3-T MRI machine (Magnetom® Skyra VD13; Siemens Healthcare GmbH, Erlangen, Germany). This enabled simultaneous imaging of the lesion, laser probe location, and thermometry for real-time monitoring of tissue ablation (Fig. 4 a and b). A 3-step checklist for safety in the intraoperative MRI suite [8] was applied. Following the last step on the list, the patient was transferred to the adjacent suite, one door separating from the operation room, and positioned in the scanner.

The laser applicator was connected with an ethernet cable to the physician workstation in the MRI control room. A three-dimensional preablation MRI scan was acquired as a reference imaging. From this image, the correct target position of the applicator was confirmed, and appropriate treatment planes were identified. Thereafter, a test dose was delivered. The preferred thermal plain images were selected, and temperature checkpoints identified. High-temperature limits were set near the tip of the applicator, whereas low-temperature limits were set at the borders of the target area and near critical structures. Real-time thermal damage was estimated and MRIgLITT was delivered (Fig. 4a and b).

A postprocedural MRI scan (Fig. 1b) to evaluate the extent of ablation was obtained and merged with the preprocedural images. Based on the planned trajectory, the correctness of the laser applicator insertion and tip position and accuracy of the thermal therapy were verified. Following completion of the operation, the applicator and anchor were withdrawn as one piece to avoid shearing off portions of the catheter. Thereafter, the skin incision was closed by a single stich 3.0-prolene non-absorbable suture under sterile conditions. Finally, the patient was awakened from general anesthesia, extubated, and transferred to the neurosurgical intermediate care unit for postoperative monitoring.

## Discussion

With recent CE marking of MRIgLITT, its availability and popularity especially in Europe are quickly expanding. There is now an increasing need for alternative and novel adjustments to enable individual use of the method and to allow more centers to treat patients with MRIgLITT according to the neurosurgeon’s personal experience, familiarity and preference, the institution’s own facilities, recourses, and infrastructure, and the local health care systems.

Surgical and technical variations and nuances for MRIgLITT are manifold and comprise both frame-based [2, 4, 5] and frameless [1, 4, 5, 7] approaches, although most of the previous studies described the use of a frame [4]. All of these settings seem to allow laser applicator placement with an acceptable accuracy, yet sufficient corresponding data and comparative studies are widely lacking. No single system has provided clear benefit over another and each has its inherent advantages and disadvantages [5]. However, frame-based stereotaxy seems to be associated with lower complication rates and reduced numbers of applicator placements when compared to frameless methods [1].

## Setting advantages and disadvantages

The RM apparatus is relatively heavy and cumbersome, its montage, adjustment, and application are complex and require some experience, and the system is based on the more unusual translational principle and polar coordinate system. Nevertheless, image quality is excellent and artifacts on neuroimages rarely occur with the RM apparatus. Moreover, due to its construction and 3-point fixation on the base ring, it has an extremely high mechanical stability and target accuracy.

With the help of the advanced target point simulator, which allows to detect miscalculations, maladjustments, and even bending of the instruments and deviations of their axis, the adjusted coordinates can be directly checked [3, 6]. Apart from the Zamorano-Dujovny (ZD) bow used with the same simulator and base ring, and the Brown-Roberts-Wells (BRW) and Cosman-Roberts-Wells (CRW) frames with their own target verification simulators, the ring-based RM stereotactic system is the only one worldwide with this so-called phantom. Theoretical planning from the software tools can be directly measured and verified according to the real-world geometric environment ensuring optimal precision and maximal safety. It allows safety checks to ensure instruments are applied and functioning properly and within acceptable tolerances prior to neurosurgical intervention.

Although in principle any target within the brain can be reached from any entrance point with the RM device [3], access in the posterior and inferior part of the skull and especially in the suboccipital region bears some intrinsic limitations in the frame positioning. Consequently, this might require meticulous stereotactical planning, adjustments of ring and bow fixation, and thus modifications of the procedures. Issues are not as readily apparent with several of the lower-profile frame-based and frameless approaches.

Additionally, the drill guide kit and centering screwing aid applied in this case can significantly contribute to the stability and accuracy of the entire setting. They help avoid drill deviation especially when drilling more tangentially to the skull and support precise insertion of the laser applicator into the bone anchor. No similar and comparable system is described and available. The kit can also be utilized for the ZD bow (Inomed, Medizintechnik GmbH, Emmendingen, Germany) as well as for the Leksell® G and the Vantage™ stereotactic systems (Elekta Instruments AB, Stockholm, Sweden).

Although at our institution further stereotactic options such as frame-associated neurosurgical robots, frameless arm-based systems, a mobile CT, and the intraoperative MRI are available, the RM apparatus is routinely used. Rationale for selecting the RM apparatus include the availability of a phantom, an accuracy within 1 mm (unpublished data), reduced need for additional equipment, and a shorter procedure time compared to some of the other settings. When compared to other laser applicator insertion systems such as frameless articulated arms (Varioguide™; Brainlab AG, Feldkirchen, Germany and Vertek®; Medtronic Inc., Minneapolis, MN, USA) and less heavy frame-based options such as the ZD, Leksell, and CRW (Integra LifeSciences, Plainsboro, NJ, USA) system, the setting described with its highly stable and accurate RM frame combined with the rigid drill guide kit maximally reduces the risk of bone anchor dislocation and inadequate laser probe placement. Moreover, the target point simulator allows direct control of correctness of adjusted coordinates. Although less stable, the frameless approaches and trajectory-guided skull-mounted mini-frames such as the ClearPoint® (MRI Interventions Inc., Irvine, CA, USA) and STarFix™ (FHC Inc., Bowdoin, ME, USA) system along with some of the frame-based alternatives might allow the surgeon a higher flexibility to insert the laser applicator. The use of the CRW frame with the Visualase® kit requires calculation of an offset to accommodate the bone anchor in cases of longer trajectories. Mini-frame platforms can be used to perform MRIgLITT entirely in the MRI suite. Frameless neuronavigation-based systems and some robotic platforms such as the ROSA ONE® Brain (Zimmer Biomet Robotics, Montpellier, France) can be used completely without frames especially for cases of larger lesions and longer trajectories but require anatomical registration in-bone fiducials that have to be inserted into and later removed from the skull. We alternatively apply the drill guide kit with the Leksell® G frame combined with a neuromate® robotic system (Renishaw, Gloucestershire, UK) using individual self-constructed tubes.

Especially as the drill has a heavy handpiece and can produce high vibrations during drilling, a maximum of stability is desired. This stability is what the implemented system provides beyond other set-ups described previously, apart from robotic systems. What is more is it allows centers already utilizing the RM apparatus to directly perform MRIgLITT and provides an excellent alternative to other methods of brain biopsy and laser probe placement. In our experience, the high stability and precision of the combined targeting system and insertion kit predispose for the use in laser applicator placement and MRIgLITT.

## Study limitations

The present report is limited to just a single MRIgLITT application. As such, further comparative assessments are desired. However, the purpose was not to evaluate the advantages and disadvantages of competing MRIgLITT approaches nor to decidedly compare our findings to other stereotactic systems and laser ablation techniques. We focused specifically on the utility of the setting described and could elucidate that the combination of an RM system combined with the drill guide kit is feasible and a useful tool for stereotactic frame-based biopsy and MRIgLITT. Given the importance of the highly emerging and rapidly increasing method of MRIgLITT, we were encouraged to describe our setting at this early stage and felt that it was important to share our preliminary experience.

## Conclusion

We present the first report on the application of a traditional RM stereotactic system combined with a newly developed drill guide kit for the use in MRIgLITT. This setting and procedure proved to be stable, accurate, and reliable for both stereotactic frame-based biopsies and in combination with MRIgLITT when properly applied.

## Abbreviations

BRW: Brown-Roberts-Wells
CE: Conformité Européenne
CRW: Cosman-Roberts-Wells
CT: Computed tomography
EU: European Union
FDA: Food and Drug Administration
MRI: Magnetic resonance imaging
MRIgLITT: Magnetic resonance imaging-guided laser interstitial thermal therapy
RM: Riechert-Mundinger
T: Tesla
USA: United States of America
WHO: World Health Organization
ZD: Zamorano-Dujovny
